# Polyphenol supplementation alters intramuscular apoptotic signaling following acute resistance exercise

**DOI:** 10.14814/phy2.13552

**Published:** 2018-01-22

**Authors:** Jeremy R. Townsend, Jeffrey R. Stout, Adam R. Jajtner, David D. Church, Kyle S. Beyer, Joshua J. Riffe, Tyler W. D. Muddle, Kelli L. Herrlinger, David H. Fukuda, Jay R. Hoffman

**Affiliations:** ^1^ Exercise and Nutrition Science Graduate Program Lipscomb University Nashville Tennessee; ^2^ Institute of Exercise Physiology and Wellness University of Central Florida Orlando Florida; ^3^ Human Performance Laboratory Kent State University Kent Ohio; ^4^ Kemin Foods L.C. Des Moines Iowa

**Keywords:** Black tea extract, EGCG, green tea extract, muscle damage, theaflavins

## Abstract

The purpose of this study was to examine the effects of 28‐days of supplementation with an aqueous proprietary polyphenol blend (PPB) sourced from *Camellia sinensis* on intramuscular apoptotic signaling following an acute lower‐body resistance exercise protocol and subsequent recovery. Untrained males (*n* = 38, 21.8 ± 2.7 years, 173.4 ± 7.9 cm, 77.6 ± 14.6 kg) were randomized to PPB (*n* = 14), placebo (PL;* n* = 14) or control (CON;* n* = 10). Participants completed a lower‐body resistance exercise protocol comprised of the squat, leg press, and leg extension exercises. Skeletal muscle microbiopsies were obtained from the vastus lateralis preexercise (PRE), 1‐h (1HR), 5‐h (5HR), and 48‐h (48HR) post‐resistance exercise. Apoptotic signaling pathways were quantified using multiplex signaling assay kits to quantify total proteins (Caspase 3, 8, 9) and markers of phosphorylation status (JNK, FADD, p53, BAD, Bcl‐2). Changes in markers of muscle damage and intramuscular signaling were analyzed via separate repeated measures analysis of variance (ANOVA). Change in Bcl‐2 phosphorylation at 1H was significantly greater in PL compared to CON (*P* = 0.001). BAD phosphorylation was significantly elevated at 5H in PL compared to PPB (*P* = 0.015) and CON (*P* = 0.006). The change in JNK phosphorylation was significantly greater in PPB (*P* = 0.009), and PL (*P* = 0.017) compared to CON at 1H, while the change for PL was elevated compared to CON at 5H (*P* = 0.002). A main effect was observed (*P* < 0.05) at 1H, 5H, and 48H for p53 and Caspase 8, with Caspase 3 and Caspase 9 elevated at 48H. These data indicate that chronic supplementation with PPB alters apoptotic signaling in skeletal muscle following acute muscle‐damaging resistance exercise.

## Introduction

Apoptosis, or programmed cell death, is an essential physiological process regulating cellular development and homeostasis often associated with advanced stages of inflammation and disease. Cellular apoptotic signaling is regulated by several key intracellular and extracellular pathways. The intrinsic apoptotic pathway involves the mitochondria as a focal point of cellular apoptotic events (Wolter et al. 1997; Moll and Zaika 2001). Upon mechanical and biochemical cellular disruption, stress‐activated protein kinases such as c‐Jun N‐terminal kinase (JNK), are upregulated and initiate a cascade of cellular processes (Hamada et al. 1998; MacKenna et al. 1998; Boppart et al. 1999). While JNK has demonstrated a variety of roles in response to cellular stress including inflammation and hypertrophy, it also has been implicated in activation of Bcl‐2 proteins (Yang et al. 1997; Yu et al. 2004; Wei et al. 2008). Bcl‐2 family (Bcl‐2, Bax, and BAD) regulate inner and outer mitochondrial membrane permeability which, if disrupted, results in mitochondrial release of caspase protease activators, inducing cellular death (Wolter et al. 1997; Yang et al. 1997; Kroemer et al. 2007).

External activation of apoptotic events is thought to result from elevated cytokine and glucocorticoid secretion initiated by a ligand binding death receptor to Fas resulting in fas associated death domain (FADD) activation (Ashkenazi 2002). This binding appropriates procaspase‐8 accumulation at the plasma membrane and induces caspase‐8 activation (Yang et al. 2005). Upon activation, caspase‐8 initiates a cascade of events eventually resulting in DNA fragmentation and cellular death (Li et al. 2001). Activation of apoptotic processes in skeletal muscle is associated with disease processes and is interconnected with myonuclear loss, DNA fragmentation, fiber atrophy, and protein degradation (McArdle et al. 1999; Brooks and Myburgh 2014; Tower 2015). However, muscular contractions and physical exertion result in mechanical and metabolic stress, activating apoptotic signaling pathways similar to other forms of cellular stress (Sandri et al. 1995, 1997; Podhorska‐Okolow et al. 1998, 1999; Paulsen et al. 2012). Eccentric muscular damage protocols have been shown to upregulate caspase and bcl‐2 family activity (Stupka et al. 2000; Willoughby et al. 2003b; Kerksick et al. 2008), however, there is relatively little data regarding the time course of skeletal muscle apoptotic activity following resistance exercise in humans.

Nutritional interventions to attenuate inflammation, apoptosis or muscle degradation is of interest to active individuals with the belief that they may directly or indirectly benefit muscular recovery and subsequent performance. Moreover, polyphenols have been reported to protect against exercise‐induced oxidative stress (Panza et al. 2008; Jówko et al. 2011), attenuate indices of muscle damage (Herrlinger et al. 2015), and facilitate recovery following strenuous exercise (Connolly et al. 2006; Arent et al. 2010; Sumners et al. 2011). Specifically, *Camellia sinensis* (tea) contains natural polyphenols, catechins, and theaflavins which have shown to alter apoptotic activity in vitro (Hofmann and Sonenshein 2003; Leone et al. 2003; Hou et al. 2004; Halder et al. 2007; Lahiry et al. 2009). While a body of literature exists examining the effects of polyphenol supplementation on markers of inflammation (Herrlinger et al. 2015; Jajtner et al. 2016), oxidative stress (Panza et al. 2008; Jówko et al. 2011), and cancer progression (Hofmann and Sonenshein 2003; Leone et al. 2003; Hou et al. 2004), limited research has explored the influence of tea polyphenols on skeletal muscle apoptosis following dynamic resistance exercise. Thus, the purpose of this study was to observe the time course effects of an acute, high volume lower‐body resistance exercise protocol and subsequent recovery on intramuscular apoptotic signaling. Furthermore, we sought to evaluate the effects of 28‐days of supplementation with a polyphenol blend on resistance training induced apoptotic signaling in untrained males.

## Methods

### Participants

Thirty‐eight healthy recreationally active men (18–35 years old) volunteered to participate in this study. Participants were randomly assigned to one of three groups. The first group consumed 2 g per day of a proprietary polyphenol blend (PPB) supplement; the second group consumed 2 g per day of a placebo (PL) and the third group served as control (CON), with no supplement or exercise. The anthropometric performance and compliance characteristics of the 38 participants are displayed in Table 1. The research protocol was approved by the New England Institutional Review Board prior to participant enrollment. Following an explanation of all procedures, risks, and benefits, each participant provided his informed written consent before completing any testing. For inclusion in the study, participants had to participate in less than 3 h of planned exercise per week, have a body mass index of 18.0–34.9 kg/m^2^, be free of physical limitations, and be willing to maintain a constant diet while abstaining from tea, alcohol, and additional dietary supplements.

**Table 1:** Participant characteristics

Characteristic	Polyphenol blend (PPB)	Placebo (PL)	Control (CON)
*n*	14	14	10
Age (y)	21.6 ± 2.5	21.5 ± 2.5	22.5 ± 3.4
Height (cm)	170.9 ± 5.4	176.1 ± 4.9	173.3 ± 12.6
Weight (kg)	70.9 ± 7.9	84.2 ± 16.3	77.8 ± 16.4
BMI (kg·m^−1^)	24.3 ± 2.6	27.0 ± 4.4	25.7 ± 3.5
Squat 1RM (kg)	107.1 ± 14.4	109.7 ± 31.3	123.0 ± 32.1
Leg Press 1RM (kg)	158.4 ± 39.8	161.7 ± 60.6	196.6 ± 76.5

Data are presented as mean ± SD.

### Study design

For this randomized, placebo‐controlled trial, all participants reported to the Human Performance Laboratory for 5 days of testing. In advance of the first day of testing, PPB and PL completed a 28‐day supplementation protocol. Before the acute resistance exercise protocol, participants reported to the lab and underwent 1‐repetition maximum (1‐RM) testing of squat, leg press and leg extension exercises, all of which occurred at least 3 days to 1 week before the start of the resistance exercise protocol. On the day of the acute resistance exercise bout, participants arrived in the lab after following a 10‐h fast and provided a resting blood sample and muscle biopsy (PRE). After blood samples had been obtained, participants were provided a small breakfast bar (Cal: 190, CHO: 19 g, Protein: 7 g, Fat: 13 g). Following breakfast consumption, CON rested for 1 h, while PPB and PL began the acute, intense exercise protocol. Participants provided blood samples immediately post‐ (IP), 1‐h (1H) and 5‐h (5H) postexercise (PPB and PL) or 1‐h rest (CON). In addition, muscle biopsies were obtained at 1H and 5H postexercise. Following the 1H muscle biopsy, participants were provided with a light meal (Cal: 250, CHO: 34 g, Protein: 14 g, Fat: 6 g). Participants returned to the lab in a fasted state 24‐h (24H) and 48‐h (48H) later for resting blood samples and an additional muscle biopsy at 48H. Adherence to the fasting and diet criteria (no tea, alcohol or additional supplements) was confirmed each morning with participants as they arrived for testing.

### Supplementing protocol

Both the PPB and PL groups completed daily supplementation for 28 days. The PPB group consumed a proprietary polyphenol blend of water‐extracted green and black tea (*Camellia sinensis*) containing at minimum 40% total polyphenols, 1.3% theaflavins, 5–8% epigallocatechin‐3gallate (EGCG), 7–13% caffeine, and 600 ppm manganese (Kemin Foods, L.C., Des Moines, IA, USA). The PL group consumed microcrystalline cellulose in capsules of similar shape, color, and size. All products were tested for toxins including heavy metals and pesticides, by an independent third‐party.

Briefly, participants reported to the Human Performance Laboratory 3–5 days per week to receive the supplements. Participants took one dose (1 g PPB or PL) under the supervision of a member of the research team and were provided their remaining prescribed doses in individual containers (1 g PPB or PL) for each additional time point. Participants consumed two 1000 mg doses per day for a total of 2000 mg per day of either PPB or PL daily. Participants were asked to return all empty containers upon their next visit to the laboratory. Participants who did not maintain 80% compliance in each phase (28 days of supplementation or during the acute exercise protocol) were removed from the analysis.

### Nutritional analysis

Participants were provided a food log for the 2 days prior to testing, as well as each day of the testing protocol (for a total of 6 days). Participants were asked to consume the same diet prior to all testing periods. The USDA Nutritional Database (US Department of Agriculture, Beltsville, MD) was used to analyze total calories, carbohydrates, protein, and fat. Values are represented as the average intake across the 6 days of data collection.

### 1‐Repetition maximum testing

Direct measurement of one repetition maximal strength (1‐RM) was completed on the squat and leg press exercises, while a predicted 1‐RM was performed on the leg extension exercise. All participants completed a standardized warm‐up, consisting of 5 min on a cycle ergometer against a self‐selected resistance, 10 body weight squats, 10 walking lunges, 10 dynamic hamstring stretches, and 10 dynamic quadriceps stretches. All 1‐RM testing was completed as previously described (Hoffman 2006). Briefly, each participant completed two warm‐up sets consisting of 5–10 repetitions and 3–5 repetitions at approximately 40–60% and 60–80% of his perceived maximum, respectively. Each participant then performed up to five subsequent trials to determine his 1‐RM with 3–5 min of rest between each set.

During the squat exercise, participants placed a safety squat bar (Power Lift, Jefferson, IA, USA) across their shoulders and descended to the parallel position, where the greater trochanter of the femur reached the same level as the knee. Participants then ascended to a complete knee extension. Leg press was completed with the participant sitting in a reclined position, with their legs extended. Participants were asked to lower the weight until the lower leg and femur created a 90° angle. Participants were then asked to press the weight up. Participants that were unable to complete the repetition or maintain proper range of motion were given one additional opportunity. If they were still unable to perform the exercise correctly, the last completed weight was recorded as the 1‐RM.

For the leg extension exercise, participants were placed in a seated position and asked to extend their legs straight out in front of them. Participants were asked to perform as many repetitions as possible, and the resulting repetitions and weight used were applied to a prediction equation (Brzycki 1993). If more than 10 repetitions were performed, the weight was increased, and the participant repeated the measure 3–5 min later. All testing was observed by a certified strength and conditioning specialist to monitor adherence to form.

### Acute exercise protocol

Only PPB and PL completed the acute exercise protocol, while CON rested for an hour. The exercise protocol designed to cause muscle damage in previously untrained individuals was preceded by a light warm‐up as described above. Following the light warm‐up, participants completed a resistance exercise session that consisted of six sets of 10 repetitions of the squat, as well as four sets of 10 repetitions of the leg press and leg extension exercises. All exercises were completed at 70% of the subjects previously determined 1‐RM with 90 sec of rest between each set. Participants were provided with assistance if they were unable to complete 10 repetitions on their own, and weight for the subsequent set was reduced. All testing sessions were observed by a certified strength and conditioning specialist to monitor adherence to exercise technique.

### Blood sampling

Blood samples were obtained at six‐time points throughout the study (PRE, IP, 1H, 5H, 24H, 48H). The PRE, IP and 1H blood samples were obtained using a Teflon cannula placed in a superficial forearm vein using a three‐way stopcock with a male Luer lock adapter and a plastic syringe. The cannula was maintained patent using an isotonic saline solution (Becton Dickinson, Franklin Lakes, NJ, USA). PRE and 1H blood samples were obtained following a 15‐minute equilibration period, while IP blood samples were taken within 5‐min of exercise cessation. The remaining time points (5H, 24H, 48H) were obtained using a single‐use disposable needle with the subject in a supine position for at least 15 min before sampling. All blood samples were collected into untreated 10 mL Vacutainer^®^ tubes. The blood was allowed to clot at room temperature for 30 min and subsequently centrifuged at 3000*g* for 15 min. The resulting serum was placed into separate micro‐centrifuge tubes and frozen at −80°C for later analysis.

### Markers of muscle damage

Serum concentrations of myoglobin (MG) were obtained via enzyme‐linked immunosorbent assay (ELISA) (Calbiotech, Spring Valley, CA, USA), while CK was analyzed using a commercially available kinetic assay (Sekisui Diagnostics, Charlottetown, PE, Canada), per manufacturer's instructions. To limit inter‐assay variability, all samples for a particular assay were thawed once and analyzed by the same technician using a BioTek Eon spectrophotometer (BioTek, Winooski, VT, USA). All samples were analyzed in duplicate with a mean coefficient of variation of 7.57% for MG and 3.66% for CK.

### Fine needle skeletal muscle biopsy procedure

Fine needle muscle biopsies were performed on the vastus lateralis muscle of the participant's dominant leg using a spring‐loaded, reusable instrument with 14‐gauge disposable needles and a coaxial introducer (Argon Medical Devices Inc., Plano, TX, USA). To confirm the site of the muscle belly for placement of the biopsy needle, ultrasonography was performed using a linear probe (LOGIQ P5; General Electric, Wauwatosa, WI) to optimize spatial resolution. The probe was coated with a water‐based conduction gel (Aquasonic 100 ultrasound transmission gel; Parker Laboratories, Inc., Fairfield, NJ) and positioned on the surface of the skin to provide acoustic contact without depressing the dermal layer. Following local anesthesia with 2 mL of 1% lidocaine applied into the subcutaneous tissue, a small incision to the skin was made, and an insertion cannula was placed perpendicular to the muscle until the fascia was pierced (Townsend et al. 2016b). The biopsy needle was inserted through the cannula, and a muscle sample was obtained by the activation of a trigger button, which unloaded the spring and activated the needle to collect a muscle sample. Each muscle sample was removed from the biopsy needle using a sterile scalpel and was subsequently placed in a cryotube, rapidly frozen in liquid nitrogen, and stored at −80°C. All muscle biopsies were performed by the same licensed medical physician.

### Intramuscular apoptotic signaling analysis

Multiplex assays were used to quantify the phosphorylation status of proteins specific to the NF‐*κ*B signaling pathway using a multi‐plex plate reader (MAGPIX^®^; Luminex, Austin, TX, USA) and a multiplex signaling assay kit (EMD Millipore, Billerica, MA, USA) according to manufacturer's guidelines. Samples were analyzed for the phosphorylated status of FADD (Ser194), BAD (Ser112), Bcl‐2 (Ser70), p53 (Ser46), and JNK (Thr183/Tyr185). In addition, active Caspase 3 (Asp175), Caspase 8 (Asp384), and Caspase 9 (Asp315) were quantified. Total protein quantification was conducted using a detergent‐compatible (DC) protein assay kit (Bio‐Rad, Hercules, CA, USA) so that all results could be reported as mean fluorescence intensity (MFI) based on the multiplex assay results relative to total protein content. To eliminate inter‐assay variance, all tissue samples were thawed once and analyzed in duplicate within the same assay run by a single technician. The average coefficient of variation was 11.2% for the phospho‐protein analysis.

### Statistical analysis

Prior to analysis all data were assessed to ensure normal distribution, homogeneity of variance and sphericity. If sphericity was violated, a Greenhouse Geisser correction was applied. Physical characteristics and dietary composition between both groups were examined using independent samples t‐test. Two‐way, between subjects repeated measures ANOVAs [time x group] where used to analyze all the variables. In the event of a significant *F* ratio, a one‐way, within‐subjects repeated measures ANOVA for each group and a one‐way, between subjects ANOVA at each time point with LSD pairwise comparisons were used for post hoc analysis. In addition, change scores for the intramuscular signaling proteins (PRE‐1H, PRE‐5H, PRE‐48H) were analyzed via separate one‐way ANOVAs. When appropriate, follow‐up analyses included one‐way between‐subject repeated measures ANOVAs and LSD post hoc comparisons. Significance was accepted at an alpha level of *P*≤0.05 and all data are reported as mean ± SD. Furthermore, the partial eta squared statistic was calculated for effect size for all dependent variables, and according to Green et al. (2000), 0.01, 0.06, and 0.14 were interpreted as small, medium, and large effect sizes, respectively. All data are reported as mean ± SD. Statistical analyses were performed using SPSS version 22.0 (IBM SPSS Statistics for Windows, Version 22.0. Armonk, NY: IBM Corp).

## Results

### Participant characteristics

Forty‐eight participants were initially recruited for this investigation, of which 10 were removed before analysis (PPB = 5, PL = 5) for a final of 38 participants in the analysis (Table 1). Of the 10 participants removed, four participants requested to discontinue testing (PPB = 3; PL = 1). Of the four participants that wished to discontinue, two reported unresolvable scheduling conflicts (both from PPB group), and two discontinued supplementation during the 28‐day supplementation period due to the time commitment (PPB = 1, PL = 1). Five additional participants that completed testing were removed from analysis due to lack of compliance (PPB = 2; PL = 3). Four of the five participants were removed for failure to achieve 80% compliance with supplementation (PPB = 1; PL = 3) while one did not adhere to the fasting requirements (PL group). One additional participant was removed due to a lack of sufficient tissue for analysis (PL group). There were no significant differences (*P* > 0.05) in baseline characteristics of the remaining 38 participants (Table 1). No significant differences (*P* = 0.902) were observed between groups for supplement compliance between the PL (97.9 ± 2.9%) and PPB (97.7 ± 3.8%). In addition, no significant differences (*F* = 0.173, *P* = 0.842) in dietary composition were noted between the three groups throughout the acute protocol.

### Markers of muscle damage

A significant group × time interaction (*F* = 7.202, *P* ≤ 0.001, *η* ^2^ = 0.292) was observed in changes in myoglobin concentrations. Myoglobin concentrations were significantly increased from PRE in PPB and PL at IP (*P* ≤ 0.001), 1H (*P* ≤ 0.001), and 5H (*P* ≤ 0.001). Myoglobin concentrations were not significantly elevated in CON at any time point, and there were no significant differences in myoglobin between PL and PPB at any time point.

A significant group x time interaction was observed (*F* = 5.122, *η* ^2^ = 0.232, *P* = 0.001) for CK concentrations. Significant elevations from PRE were observed in PPB at 24H (*P* = 0.001) and 48H (*P* = 0.002) while PL was also elevated at 24H (*P* = 0.006) and 48H (*P* = 0.020). Creatine Kinase values were not significantly elevated in CON at any time point. However, CK concentrations were significantly greater in PPB compared to CON at 24H (*P* = 0.005), and PPB was greater than CON (*P* = 0.010) and PL (*P* = 0.047) at 48H. While PL was significantly elevated in relation to PRE, it was not statistically different from CON at any time point.

### Effects of exercise on skeletal muscle phosphorylated FADD (Ser194) protein expression

There was no group x time interaction (*F* = 1.187, *η* ^2^ = 0.064, *P* = 0.324), however, there was a main effect for time (*F* = 6.478, *η* ^2^ = 0.156, *P* = 0.002) for FADD phosphorylation. FADD phosphorylation was increased compared to PRE at 5H (*P* = 0.003) and 48H (*P* = 0.003) for all groups combined. In addition, there were no interactions observed between change scores at any time point.

### Effects of exercise on skeletal muscle phosphorylated JNK (Thr183/Tyr185) protein expression

**Figure 1:**
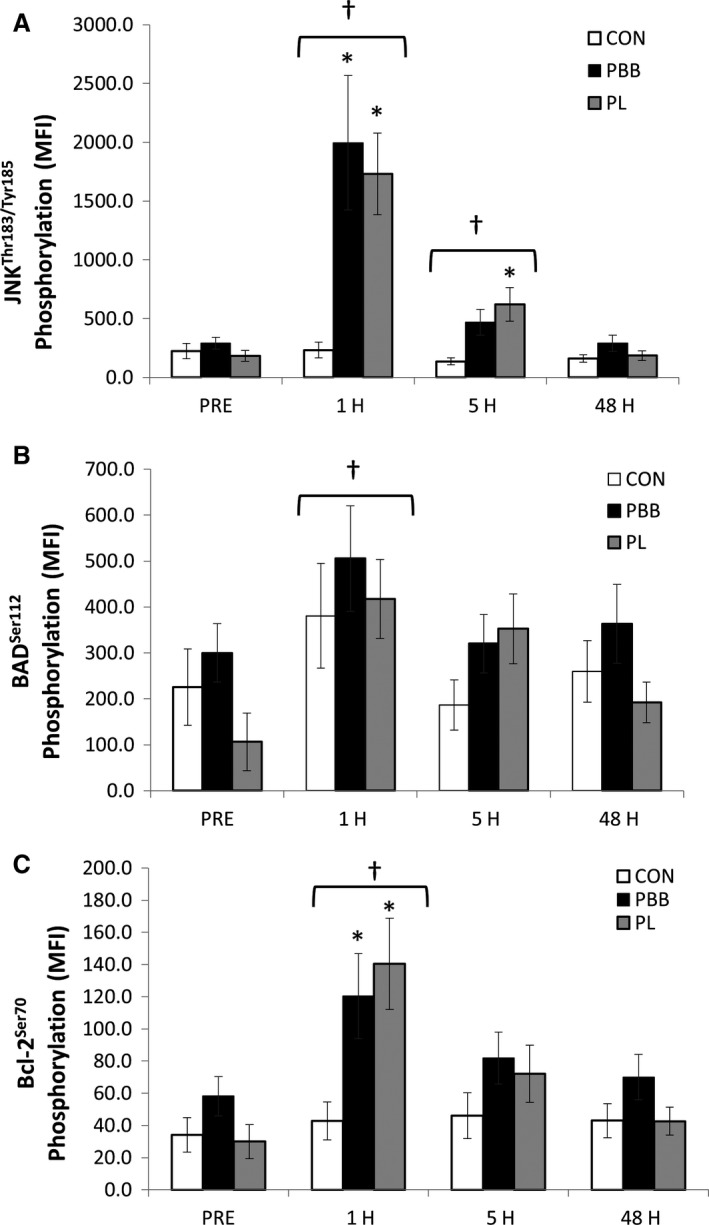
Absolute phosphorylation values for (A) JNK (Thr183/Tyr185) (B) BAD (Ser112) (C) Bcl‐2 (Ser70) following resistance exercise. CON, control; PL, placebo PPB, polyphenol blend. PRE, prior to exercise bout; 1H, 1‐h postexercise; 5H, 5 h postexercise. 48H, 48 h postexercise; MFI, mean fluorescence intensity. Data reported as mean changes ± SEM. ^†^Significant difference from PRE. *Significantly greater than CON.

**Figure 2:**
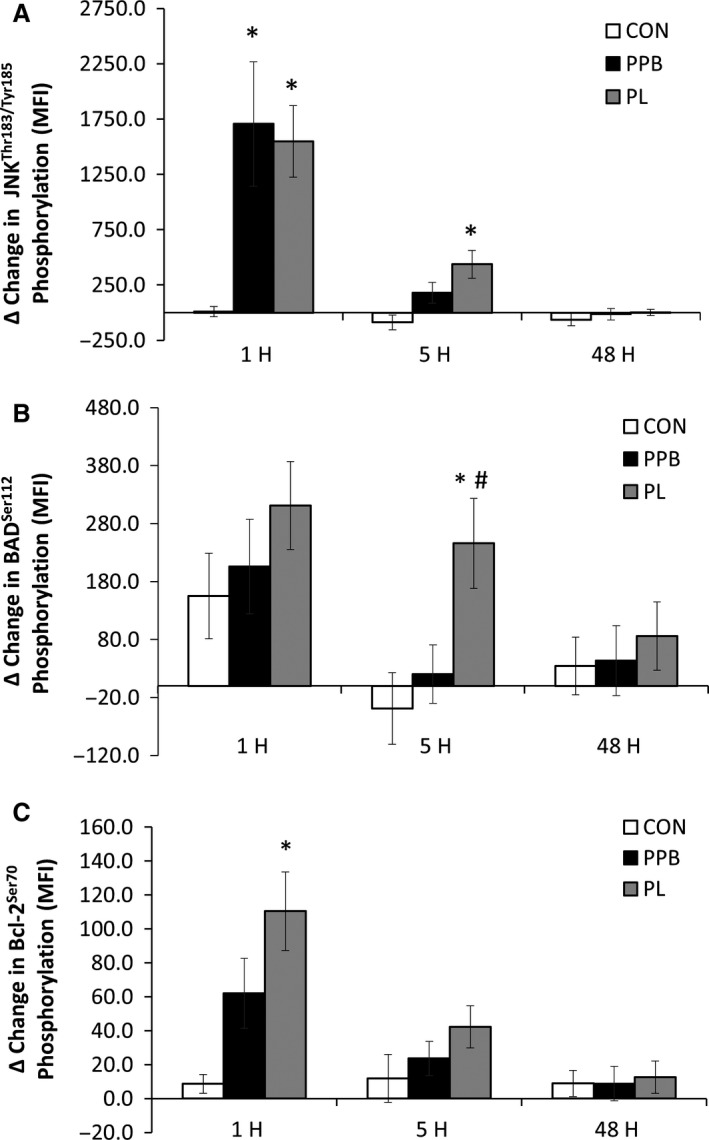
Phosphorylation delta change values for (A) JNK (Thr183/Tyr185) (B) BAD (Ser112) (C) Bcl‐2 (Ser70) following resistance exercise. CON, control; PL, placebo PPB, polyphenol blend; 1H, preexercise to 1‐h postexercise; 5H, preexercise to 5 h postexercise; 48H, preexercise to 48 h postexercise. MFI, mean fluorescence intensity. Data reported as mean changes ± SEM. *Significantly greater than CON. ^#^Significantly greater than PPB.

Absolute JNK phosphorylation values are presented in Figure 1A. Changes in JNK phosphorylation are presented in Figure 2A. A significant interaction (*F* = 4.052, *η* ^2^ = 0.351, *P* = 0.022), was observed between groups. At 1H, JNK phosphorylation was significantly elevated in PPB (*P* = 0.009) and PL (*P* = 0.024) compared to CON. JNK phosphorylation was significantly greater at 5H in PL compared to CON (*P* = 0.008). There was also a main effect for time (*F* = 18.926, *η* ^2^ = 0.351, *P* ≤ 0.001), with JNK phosphorylation significantly increased at 1H (*P* ≤ 0.001) and 5H (*P* = 0.007). Analysis of change values revealed significant interactions at 1H (*F* = 4.444, *P* = 0.019) and 5H (*F* = 5.846, *P* = 0.006). The change in JNK phosphorylation in PPB (*P* = 0.009) and PL (*P* = 0.017) was significantly greater than CON at 1H. At 5H, PL JNK phosphorylation was significantly greater (*P* = 0.002) than CON, while PPB was not significantly different from CON (*P* = 0.093). In addition, at 5H there was a trend (*P* = 0.075) for significant elevation in JNK activity in PL compared to PPB.

### Effects of exercise on skeletal muscle phosphorylated BAD (Ser112) protein expression

Absolute BAD phosphorylation values are presented in Figure 1B. Changes in BAD phosphorylation are presented in Figure 2B. There was no group x time interaction (*F* = 1.560, *η* ^2^ = 0.082, *P* = 0.193). However, there was the main effect for time (*F* = 11.609, *η* ^2^ = 0.240, *P*≤0.001), with BAD phosphorylation increased at 1H (*P* ≤ 0.001) and a trend for an increase at 5H (*P* = 0.061). Analysis of the changes in BAD phosphorylation revealed a significant interaction at 5H with PL significantly elevated in comparison to CON (*P* = 0.006) and PBB (*P* = 0.015). There were no other differences observed between groups.

### Effects of exercise on skeletal muscle phosphorylated Bcl‐2 (Ser70) protein expression

Absolute Bcl‐2 phosphorylation values are presented in Figure 1C. Changes in Bcl‐2 phosphorylation are presented in Figure 2C. There was a significant group x time interaction (*F* = 4.295, *η* ^2^ = 0.197, *P* = 0.007) with PBB (*P* = 0.045) and PL (*P* = 0.013) significantly elevated in comparison to CON at 1H. In addition, there was a main effect for time (*F* = 17.036, *η* ^2^ = 0.327, *P* ≤ 0.001) with Bcl‐2 phosphorylation increased at 1H (*P* ≤ 0.001), and 5H (*P* = 0.001). Analysis of change values revealed a significant interaction (*F* = 5.983, *P* = 0.006) for the change in Bcl‐2 phosphorylation between groups at 1H. Further analysis of the change in Bcl‐2 phosphorylation revealed PL (*P* = 0.001) was significantly elevated compared to control at 1H postexercise. No other differences were observed between groups at any other time point.

### Effects of exercise on skeletal muscle active Caspase 8 (Asp384), Caspase 9 (Asp315), and Caspase 3 (Asp175) protein expression

No significant interactions were noted in the phosphorylation changes of Caspase 8 (*F* = 1.672, *η* ^2^ = 0.087, *P* = 0.199), Caspase 9 (*F* = 0.681, *η* ^2^ = 0.037, *P* = 0.600) or Caspase 3 (*F* = 1.310, *η* ^2^ = 0.070, *P* = 0.276). However, significant main effects for time were noted in the phosphorylation of all three proteins (*F* = 4.558, *η* ^2^ = 0.115, *P* = 0.035), (*F* = 8.499, *η* ^2^ = 0.195, *P* = 0.001) and (*F* = 6.804, *η* ^2^ = 0.163, *P* = 0.002), respectively. For Caspase 8, increased phosphorylation was observed at 1H (*P* = 0.008), 5H (*P* = 0.031) and 48H (*P* = 0.006), while significant elevations were observed at 48H (*P* ≤ 0.001) only for Caspase 9 and Caspase 3. There were no significant differences in change values for Caspase 3, 8, and 9 for any of the time points.

### Effects of exercise on skeletal muscle phosphorylated p53 (Ser46) protein expression

No significant interactions (*F* = 1.898, *η* ^2^ = 0.098, *P* = 0.123) were noted in the phosphorylation changes of p53 post‐resistance exercise. However, there was a main effect for time (*F* = 3.850, *η* ^2^ = 0.099, *P* = 0.027) with p53 phosphorylation significantly increased at 1H (*P* = 0.036), 5H (*P* = 0.013), and 48H (*P* = 0.014) postexercise. Furthermore, no significant differences were observed in p53 change values at any time point.

## Discussion

The main findings of this investigation were that select markers of apoptotic signaling in skeletal muscle were upregulated in response to heavy resistance exercise and subsequent recovery in untrained males. Increased signaling was observed in both extrinsic (FADD, Caspase 8) and intrinsic (JNK, Bcl‐2, BAD, Caspase 9, p53, Caspase 3) apoptotic cellular pathways. Furthermore, we report that polyphenol supplementation resulted in attenuated intramuscular BAD phosphorylation in the initial hours of recovery from heavy resistance exercise compared to the placebo group.

JNK is known to be phosphorylated by various states of inflammation, mechanical strain, metabolic stress, and physical activity (Aronson et al. 1998; Hamada et al. 1998; MacKenna et al. 1998; Boppart et al. 1999). In this study, JNK was significantly increased compared to the control at 1H in both exercise groups, and only PL was elevated at 5H compared to the control group. These findings are largely in support of previous research regarding eccentric and dynamic resistance exercise (Aronson et al. 1998; Boppart et al. 1999; Galpin et al. 2011; Gonzalez et al. 2016). A previous investigation reported increased intramuscular JNK phosphorylation at 1H postexercise in resistance trained males following two separate resistance exercise protocols in resistance trained males (Gonzalez et al. 2016). In addition, substantial increases in JNK phosphorylation have been described during (148%) and immediately following (141%) a high intensity, power‐lifting resistance protocol (Galpin et al. 2011). JNK seems to be highly sensitive to exercise‐induced mechanical and metabolic stress as the greatest increases in phosphorylation in our study and in previous work occur near the cessation of high volume exercise (Boppart et al. 1999; Galpin et al. 2011; Gonzalez et al. 2016). At 5H postexercise, PPB appears to have attenuated intramuscular JNK phosphorylation while PL was still significantly elevated. While this is the first study to investigate polyphenol supplementation and exercise‐induced JNK activity in humans, previous in vitro work has reported enhanced JNK activity when treated with EGCG compounds (Shankar et al. 2008). As JNK elicits diverse anti‐apoptotic and cell survival mechanisms in skeletal muscle, it is currently unclear as to how attenuated JNK activity may affect muscular regeneration and repair (Boppart et al. 1999; Yu et al. 2004; Wei et al. 2008).

Initiated by JNK activity, BAD phosphorylation allows the protein to cleave from Bcl‐2 and bind with the 14‐3‐3 protein, sequestering BAD in the cytoplasm and preventing its interaction with Bcl‐2 (Ola et al. 2011). Our findings revealed increased BAD phosphorylation in the PL compared to PPB and CON at 5H postexercise while Bcl‐2 phosphorylation was elevated in both exercise groups at 1H and 5H in recovery with a trend (*P* = 0.069) toward an increase at 48H. Bcl‐2 is considered a gatekeeper anti‐apoptotic protein because it binds to the Bax protein preventing Bax accumulation and mitochondrial dismantling (Wolter et al. 1997; Yang et al. 1997; Kroemer et al. 2007). These data are in partial agreement with a previous study which found increases in Bcl‐2 activity in skeletal muscle following 100 eccentric repetitions of the knee extensors at 48H post‐resistance exercise (Kerksick et al. 2010). Kerksick et al. (2010) found no change in Bcl‐2 mRNA expression occurred at 4H and 24H following three sets of 10 repetitions of bilateral knee extensions (Yang et al. 2006). One possible reason for the discrepancy between these findings is that we utilized dynamic resistance exercise, which possibly resulted in a different metabolic and mechanical stress than previous eccentric or lower volume protocols. Furthermore, our findings indicated that PPB supplementation attenuated the increase in Bcl‐2 phosphorylation at 1H post‐resistance as the change in Bcl‐2 was significantly increased in only the PL group. To date, only one other study has investigated the effects of polyphenol compounds on resistance exercise‐induced apoptosis. In that study, Kerksick and colleagues observed no differences between 14 days of *N*‐acetyl‐cysteine (NAC) or EGCG supplementation and intramuscular Bcl‐2 levels at any time point following exercise (Kerksick et al. 2010) and no differences were observed between EGCG and placebo for the Bax:Bcl‐2 ratio, which serves as an indicator of apoptotic protein accumulation (Yang et al. 2006; Wei et al. 2008; Kerksick et al. 2010). While there is a gap in the literature regarding the effects of polyphenol supplementation on skeletal muscle apoptosis, blunted Bcl‐2 activity as a result of polyphenol administration has been reported in other cell types (Shankar and Srivastava 2007; Tsang and Kwok 2010). Specifically, ECGC has been reported to regulate apoptosis by downregulating Bcl‐2 activity in HepG2 cells, inducing apoptosis of the malignant cells (Tsang and Kwok 2010). However, the physiological significance of polyphenol‐induced attenuation of Bcl‐2 phosphorylation following exercise‐induced cellular stress is less understood. In addition, as Bcl‐2 has recently been suggested to play a significant role in exercise adaptations by regulating glucose homeostasis, more research is needed to determine the chronic effects of polyphenols and exercise training.

p53 has been reported as a tumor suppressor protein, possessing the ability to inhibit mitochondrial‐mediated cell death while also promoting and regulating mitochondrial biogenesis (Donahue et al. 2001; Moll and Zaika 2001; Saleem et al. 2009). Upon phosphorylation, p53 translocates from the nucleus to the mitochondria, thus promoting mitochondrial biogenesis by preventing p53 suppression of PGC‐1*α* (Saleem and Hood 2013). Previous animal studies have observed decreased markers of mitochondrial content, impaired contractile activity and decreased running performance in p53 knockout mice compared to wild‐type animals (Park et al. 2009; Saleem et al. 2009). In this study, change in p53 phosphorylation following resistance exercise was not different from CON in either the PL or PPB groups. While p53 activity has been shown to increase in response to muscular contraction, recent work in humans reveals that p53 phosphorylation may be dependent on the glycogen status of the muscle (Bartlett et al. 2013; Camera et al. 2015). Camera et al. (Camera et al. 2015) observed significantly elevated p53 phosphorylation following resistance exercise only in an exercise group which was glycogen depleted. Similar findings have also been reported in a study investigating high‐intensity interval training where p53 activity was threefold higher in the group with low carbohydrate availability (Bartlett et al. 2013). Therefore, the lack of exercise‐induced p53 phosphorylation in our study may be an indicator that our participants commenced exercise with adequate carbohydrate availability. Future investigations are needed to determine if glycogen availability or depletion may affect apoptosis in skeletal muscle.

The resistance exercise protocol resulted in increased caspase 8 levels during recovery at all time points, and increased caspase 3 and 9 levels at 48H with no differences observed based on treatment. Kerksick et al. (2010) similarly observed elevated caspase 3 activity at 48H following an eccentric muscle damaging protocol in both EGCG and placebo‐treated groups. In contrast, increased caspase 3 activity has been reported at 6 h and 24 h following two eccentric exercise protocols (7 sets of 10 repetitions at 150% 1RM) (Willoughby et al. 2003b). Our findings support previous work suggesting that muscle damaging activities upregulate caspase apoptotic events (Willoughby et al. 2003a,b; Yang et al. 2006; Kerksick et al. 2010). Moreover, it appears that in human skeletal muscle, tea polyphenols have limited interaction with caspase‐mediated apoptosis.

While the present study attempted to examine multiple intrinsic and extrinsic pathways involved in apoptotic cellular events, it is not without limitations. Cytochrome‐c release from the mitochondria is a key indicator of mitochondrial membrane disruption and initiates apoptotic cascades (Taylor et al. 2008; Wang and Youle 2009). Though it was outside the scope of this study, the measurement of cytochrome‐c and other markers of cellular oxidative stress (i.e., reactive oxygen species) may provide additional insight into the interconnected physiological pathways involved in apoptosis. Furthermore, our study provides a time‐course description of the immediate apoptotic events following a high‐volume resistance exercise bout, thus, it may yield limited implications on training adaptations. It has previously been reported that acute variances in apoptotic signaling do not necessarily result in altered training outcomes (Park et al. 2009). To date, supplementation with tea polyphenols has resulted in equivocal performance outcomes and training adaptations (Eichenberger et al. 2009; Jówko et al. 2011; Herrlinger et al. 2015; Kuo et al. 2015). In addition, there is a growing body of evidence to suggest that modest physiological increases in proinflammatory, apoptotic, and oxidative stress pathways may be essential and even beneficial to muscular adaptations (Hyldahl et al. 2011; Kerksick et al. 2013; Urso 2013; Townsend et al. 2016a). Nevertheless, additional aerobic and resistance training studies are needed to determine how these acute perturbations affect muscular hypertrophy and athletic adaptation.

In conclusion, this study demonstrates that high‐volume resistance exercise induces apoptotic signaling in untrained males. Furthermore, it appears that 28‐days of polyphenol supplementation modulates the acute JNK, BAD, and Bcl‐2 response to heavy resistance exercise. The regulation of apoptotic pathways is highly complex and additional work is needed to determine the physiological relevance of polyphenol‐induced alterations in apoptotic signaling following resistance exercise.

## Conflict of Interest

K. A. Herrlinger is an employee of Kemin Foods, L.C. All other authors have no actual or potential conflicts of interest to report.
